# Global research landscape and hotspots of paraptosis: a multi-database bibliometric analysis with a focused literature review

**DOI:** 10.3389/fonc.2026.1864713

**Published:** 2026-07-21

**Authors:** Jiayu Xiu, Yongkang Ma, Yuanpeng Huang

**Affiliations:** 1 Beijing University of Chinese Medicine, Beijing, China; 2 Dongzhimen Hospital, Beijing University of Chinese Medicine, Beijing, China; 3 School of Medicine, Xiamen University, Xiamen, Fujian, China; 4 Department of Oncology, Xiamen Hospital, Dongzhimen Hospital, Beijing University of Chinese Medicine, Xiamen, Fujian, China

**Keywords:** bibliometrics, cancer, endoplasmic reticulum stress, paraptosis, programmed cell death

## Abstract

**Background:**

Paraptosis is a non-canonical form of programmed cell death distinct from apoptosis and morphologically characterized by prominent cytoplasmic vacuolation. However, a systematic assessment of the overall development, research landscape, and hotspot evolution of paraptosis remains lacking. This study therefore used bibliometric methods to systematically map paraptosis-related publications, and provide an evidence-based reference for future research.

**Methods:**

Using “paraptosis” as the core search term, we systematically retrieved paraptosis-related publications from the Web of Science Core Collection, PubMed, and Scopus from database inception to December 31, 2025. Only English-language articles and reviews were included. VOSviewer, CiteSpace, R, and Excel were used to analyze and visualize publication trends, collaboration networks, journal distribution, co-citation relationships, keyword co-occurrence, clustering, and thematic evolution.

**Results:**

A total of 401 publications were included. Annual output showed an overall fluctuating upward trend, with marked growth from 2020 to 2025. China (n = 155) and Ajou University (n = 19) were the most productive country and institution, respectively, and Choi Kyeong Sook was a representative core author. Journals in this field mainly focused on cell death and related mechanisms, cancer and molecular mechanisms, and chemical biology and drug research, whereas the knowledge base was primarily grounded in basic life sciences. Sperandio S was the most frequently co-cited author (n = 356), and his publications in 2000 and 2004 were the most representative co-cited references. Paraptosis has gradually attracted systematic attention as a relatively independent topic since 2018. Major hotspots included phenotypic identification, relationships with other forms of cell death, mechanistic investigation, and cancer-related research, with mechanistic studies mainly focusing on endoplasmic reticulum stress, mitochondrial damage, and reactive oxygen species accumulation.

**Conclusion:**

Paraptosis research remains in sustained development and has gradually extended from phenotypic identification toward mechanistic elucidation and potential applications. Its value in cancer research and its potential connection with the tumor microenvironment may represent important future frontiers. Strengthening international collaboration and cross-team communication, together with high-quality applied research, may help advance the functional characterization and translational investigation of paraptosis in disease.

## 1 Introduction

Programmed cell death (PCD) is a fundamental biological process that maintains organismal development, tissue homeostasis, and the elimination of abnormal cells (1). In addition to classical forms of cell death such as apoptosis, autophagic cell death, and necroptosis, non-canonical cell death processes with distinctive morphological and molecular characteristics have attracted increasing attention. Among these, paraptosis has emerged as an important focus in recent years because of its phenotypic features that distinguish it from classical apoptosis (2). First proposed by Sperandio et al. in 2000, paraptosis was defined as a non-apoptotic form of PCD (3). It is typically characterized by prominent cytoplasmic vacuolation, with vacuole-like structures mainly derived from swollen endoplasmic reticulum (ER) and/or mitochondria. In general, paraptosis is not accompanied by classical apoptotic features such as nuclear condensation, apoptotic body formation, or caspase activation. Therefore, as a distinctive mode of PCD, the biological basis and significance of paraptosis remain to be further elucidated.

Beyond its morphological features, the intracellular regulatory processes involved in paraptosis have become important research focuses. Existing evidence indicates that paraptosis is closely associated with activation of the MAPK signaling pathway, proteostasis imbalance, ER stress, mitochondrial damage, reactive oxygen species (ROS) accumulation, and disruption of calcium (Ca^2+^) homeostasis (4, 5). AIP1/ALIX is regarded as one of the classical negative regulators of paraptosis (6). Nevertheless, unified molecular markers, core mechanisms, and key execution steps of paraptosis have not yet been fully clarified (2). The concept of paraptosis helps deepen understanding of the heterogeneity of cell death and may also provide alternative routes for cell elimination beyond classical cell death pathways, particularly in the context of cancer biology (7). Increasing studies suggest that induction of paraptosis may provide an alternative death route for cancer cells that escape apoptosis and may offer new strategies for overcoming therapeutic resistance (4, 7, 8). For example, mitocurcumin induces paraptosis in drug-resistant non-small cell lung cancer cells with restricted apoptotic responses, thereby facilitating their elimination (9). Elaiophylin preferentially kills drug-resistant ovarian cancer cells by inducing paraptosis and has shown potential to overcome resistance across multiple resistant models (10). Other studies have suggested that paraptosis induction may be associated with enhanced immunogenic cell death (ICD), indicating that its potential role may extend beyond direct cytotoxicity (11). As research on relevant regulatory molecules and interaction networks continues to progress, the value of paraptosis as a potential antitumor strategy is increasing.

In recent years, the number of paraptosis-related publications has continued to increase, and existing studies have been dominated by experimental reports and narrative reviews. Research in this field has gradually expanded from initial concept proposal and phenotypic identification to inducing factors, potential regulatory mechanisms, and possible applications. However, the developmental trajectory, research hotspots, and evolutionary trends in the paraptosis field remain insufficiently summarized. Bibliometrics, through literature statistics, knowledge-map construction, and visualization, can reveal the developmental structure, research hotspots, and frontier trends of a specific research area from a macro-level perspective (12). It therefore provides a useful framework for understanding the overall status of paraptosis research and for providing references for subsequent basic research and potential translational applications. Accordingly, this study applied bibliometric methods to systematically visualize and analyze paraptosis-related research, with the aim of providing a reference basis for future studies in this field.

## 2 Materials and methods

### 2.1 Data sources

To obtain paraptosis-related publications as comprehensively as possible, three databases were searched: the Web of Science Core Collection (WoSCC), PubMed, and Scopus. All searches and data exports were completed on the same day, to minimize potential bias caused by dynamic database updates.

### 2.2 Search strategy

“Paraptosis” was used as the core search term, and the retrieval period covered database inception to December 31, 2025. The search strategy for WoSCC was TS = (paraptosis); the search query for Scopus was TITLE-ABS-KEY (paraptosis); and the PubMed search was limited to paraptosis[Title/Abstract]. In all three databases, the searches were limited to articles and reviews published in English.

### 2.3 Data screening

#### 2.3.1 Inclusion criteria

Two researchers independently assessed the relevance of the retrieved records. Publications were included if the term “paraptosis” appeared in the title, abstract, or keywords. If the term “paraptosis” appeared only in the abstract, the abstract was reviewed in full to determine whether the publication was relevant to paraptosis research. Any disagreement during screening was resolved by a third researcher. The initial search identified 1, 299 publications, including 436 records from WoSCC, 468 from Scopus, and 395 from PubMed. WoSCC records were exported in plain-text format (.txt), Scopus records in CSV format (.csv), and PubMed records in PubMed format (.txt). Exported information included titles, authors, institutions, countries, publication years, abstracts, keywords, and references.

#### 2.3.2 Exclusion criteria

The exclusion criteria were as follows (1): duplicate publications (2); retractions, corrections, and similar records; and (3) publications with missing content or those unrelated to paraptosis research.

### 2.4 Data cleaning

**Figure 1:**
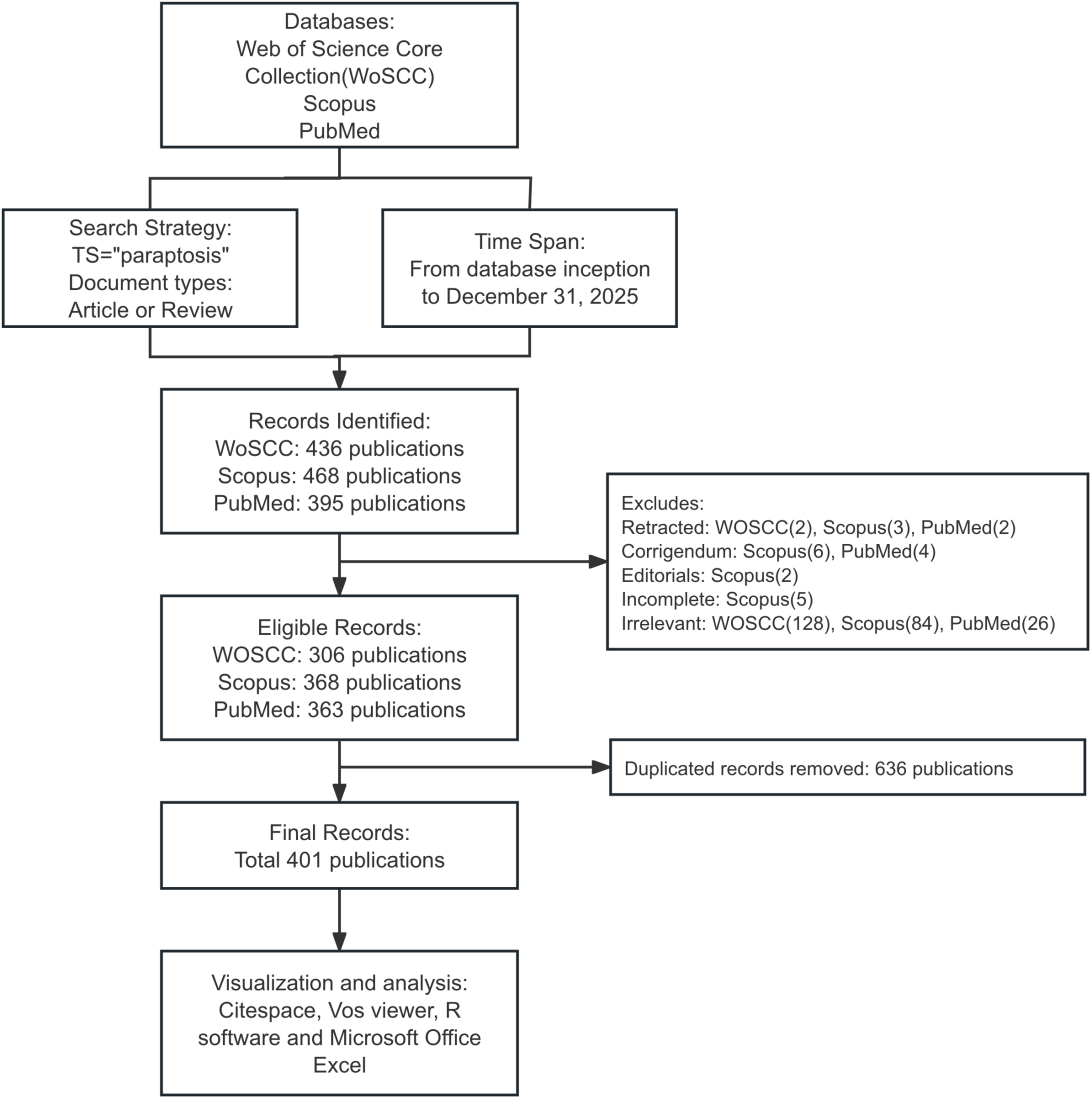
Flowchart of literature screening.

Python was used to convert Scopus CSV files into a.txt format consistent with WoSCC and PubMed files to enable unified processing. Duplicate records were first identified according to DOI. For records with missing DOIs or inconsistencies in DOI format, manual verification was performed according to title, author, publication year, and other bibliographic information. Records with “[Anonymous]” in the author field were removed, virtual institutions such as “Egyptian Knowledge Bank (EKB)” were excluded, and variant names of the same institution were standardized and merged. After deduplication, cleaning, and standardization, 401 publications were ultimately included for subsequent analyses. The literature screening process is shown in **Figure 1**.

### 2.5 Data visualization

Bibliometric analyses were conducted using the 401 publications after deduplication, cleaning, and standardization. Visual analyses were performed for annual publication trends, country and institutional collaboration networks, author collaboration networks, publishing journals and co-cited journals, co-cited authors and co-cited references, reference citation bursts, keyword co-occurrence, keyword clustering, and thematic evolution. VOSviewer, CiteSpace, R, and Excel were used for data analysis and graph construction. The key parameter settings in VOSviewer were as follows: Counting method (Full counting) and Method (LinLog/modularity). The key parameter settings in CiteSpace were as follows: time slicing (2002–2025), years per slice (1), term source (all selections), node type (one at a time), selection criterion (g-index, k = 25), and cluster labeling method (LLR). The bibliometrix package in R was used for keyword-based thematic analysis. Because network structures and node distributions differed across analytical objects, thresholds were set according to the specific analytical objectives and interpretability of each map to improve the clarity and readability of the results.

## 3 Results

### 3.1 General publication profile and annual trends

**Figure 2:**
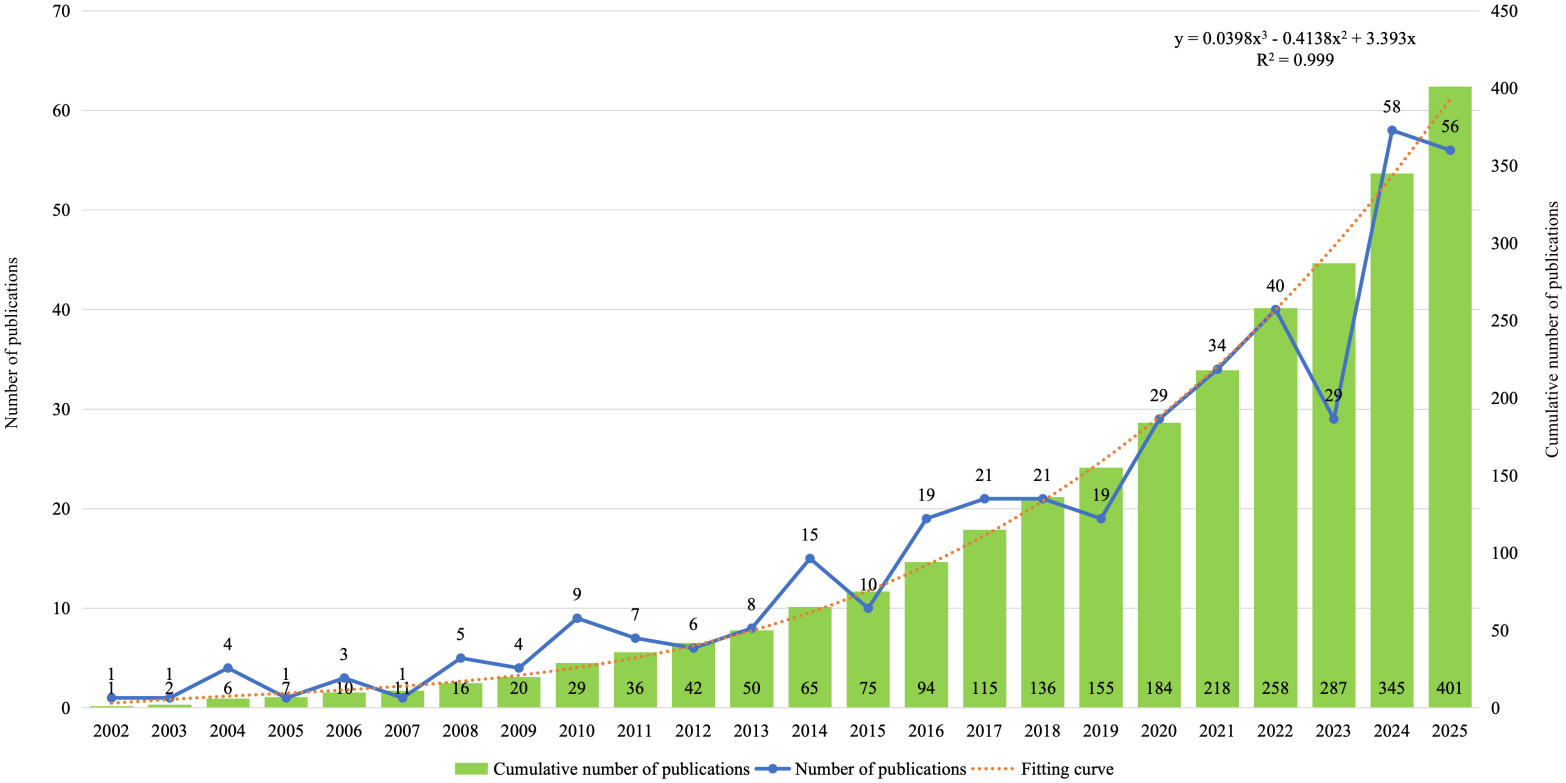
Annual and cumulative publication trends.

After screening, 401 paraptosis-related publications were included. **Figure 2** shows the annual number of publications, cumulative number of publications, and overall trend in paraptosis research. Overall, the annual output showed a fluctuating upward trend, while cumulative output continued to increase. According to the characteristics of annual output, the development of paraptosis research can be broadly divided into three stages. The period from 2002 to 2013 was the embryonic stage, during which annual output remained low and did not exceed 10 publications, suggesting that the research was still in the early exploration phase following the proposal of the concept. The period from 2014 to 2019 was the initial growth stage, during which annual output increased compared with the previous period, indicating that paraptosis research began to receive more sustained attention. The period from 2020 to 2025 was the accelerated development stage, during which publication output increased further and reached a peak in 2024, indicating markedly enhanced activity in recent years. The fitted curve showed an overall upward trend, demonstrating the continuing growth of paraptosis research.

### 3.2 Countries, institutions, and authors: research strength and collaboration patterns

#### 3.2.1 Country distribution and collaboration network

**Figure 3:**
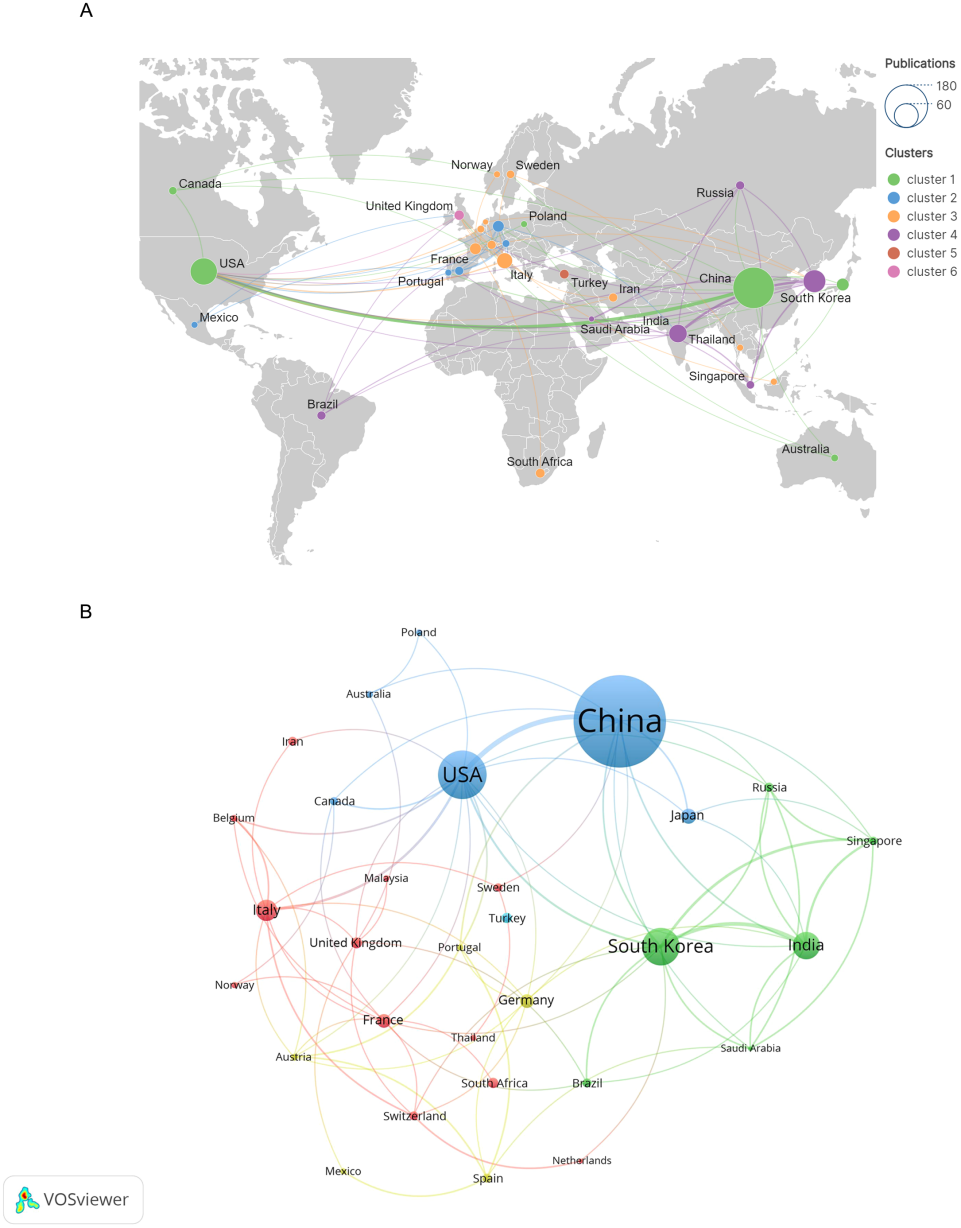
Geographic distribution of publication output by country and country collaboration network. **(A)** Geographic distribution of countries. **(B)** Country collaboration network.

**Figure 3** presents the country distribution and inter-country collaboration network of paraptosis research. Overall, the field showed a multinational research pattern, although publication output and collaboration were mainly concentrated in a small number of core countries. China and the United States were the major publishing countries in paraptosis research, with China having the largest publication output (n = 155). South Korea, India, Italy, France, Germany, and Japan were also important contributors to the field. Country-level publication frequencies are shown in **Supplementary Table 1**. The country collaboration network indicated that China, the United States, and South Korea had relatively large nodes and collaborative links with multiple countries, suggesting that they occupied relatively central positions in the international collaboration network. Some European countries formed relatively dense collaborative connections, whereas Asian countries mainly formed collaborative links around China, South Korea, and India.

#### 3.2.2 Institutional collaboration network and highly productive institutions

**Figure 4:**
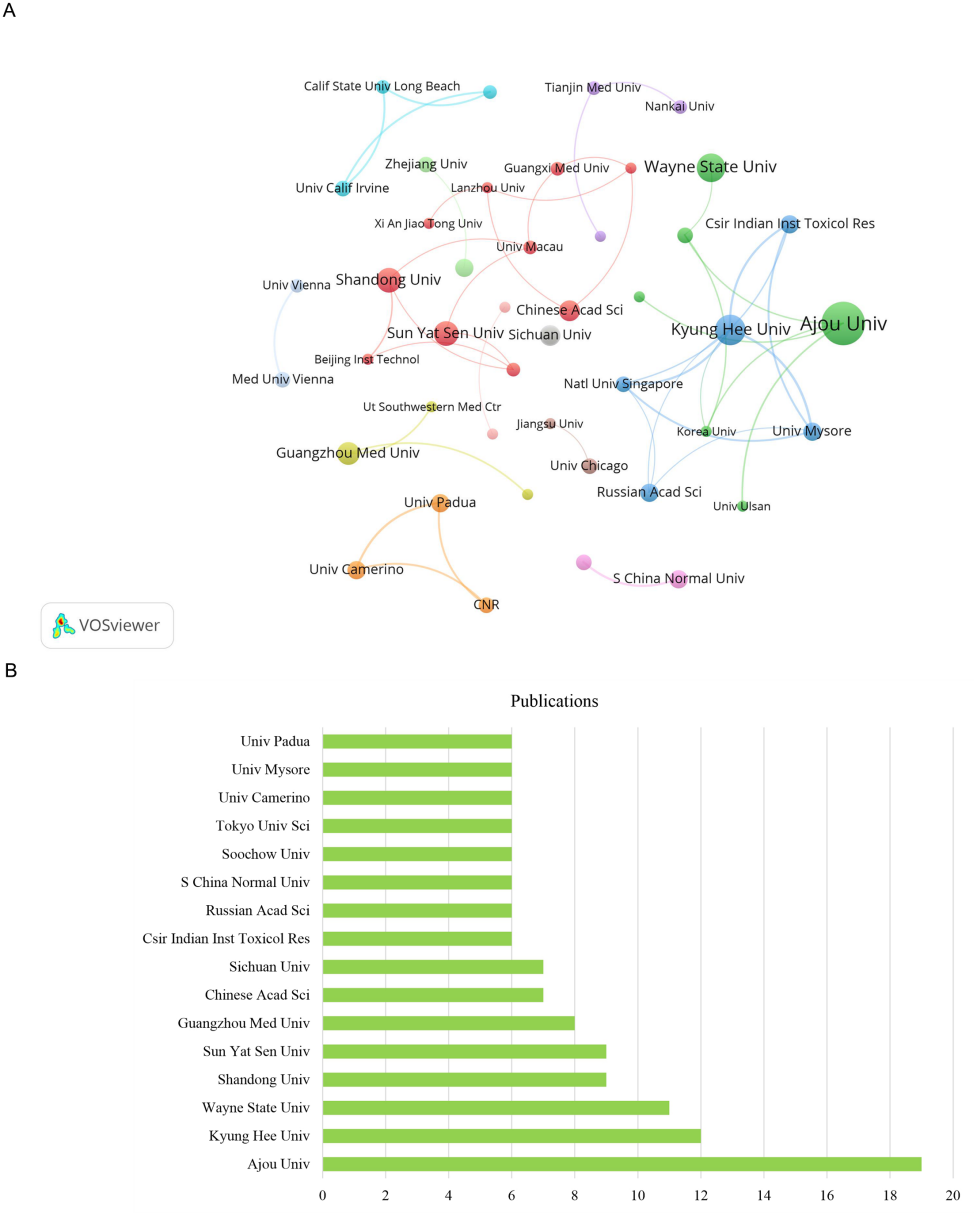
Institutional collaboration network and distribution of highly productive institutions. **(A)** Institutional collaboration network. **(B)** Top 10 institutions by publication output (including ties; 16 institutions in total).

**Figure 4** illustrates institutional collaboration and the distribution of highly productive institutions in paraptosis research. Overall, the institutional collaboration network showed clustering characteristics, and research output was mainly concentrated in a limited number of institutions. From the collaboration network (**Figure 4A**), several collaborative clusters were formed among institutions. Ajou University, Kyung Hee University, Shandong University, and Sun Yat-sen University had relatively large nodes and multiple links, suggesting relatively active collaborative relationships in this field. In terms of publication output (**Figure 4B**), Ajou University ranked first with 19 publications, followed by Kyung Hee University (12 publications) and Wayne State University (11 publications). The remaining highly productive institutions each published fewer than 10 articles, indicating a certain concentration of institutional output, although the publication gap among leading institutions was not yet pronounced.

#### 3.2.3 Author collaboration network and characteristics of core authors

**Figure 5:**
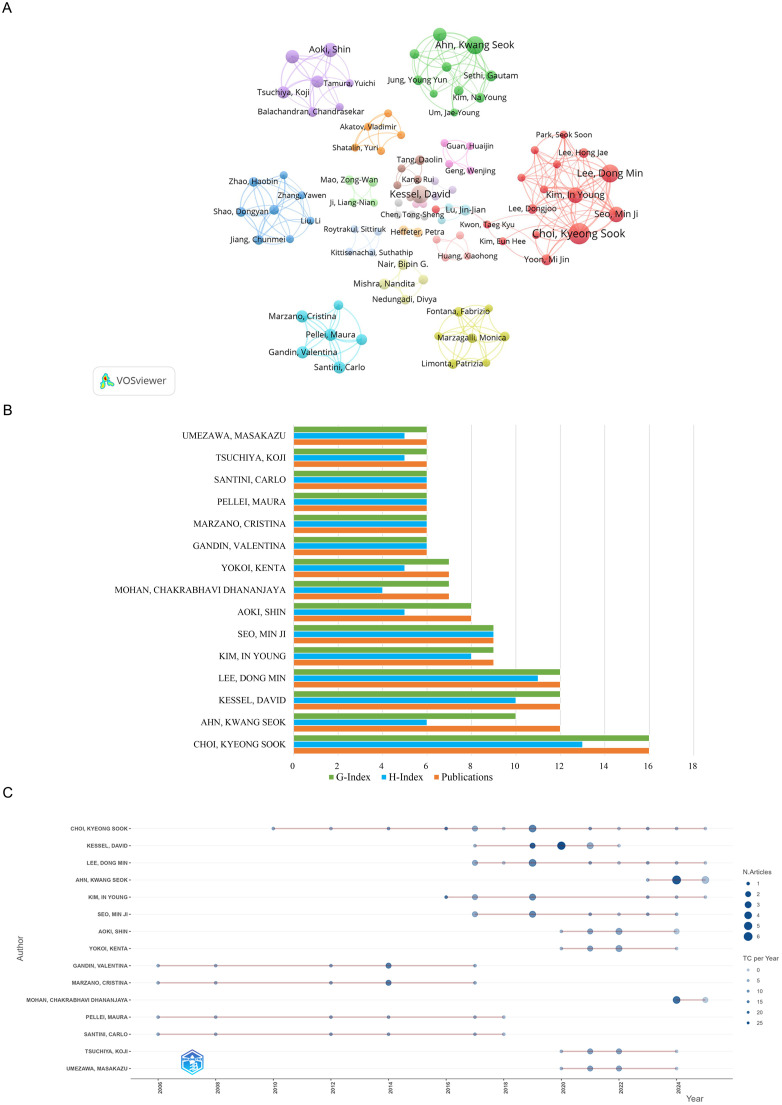
Author collaboration network and characteristics of core authors. **(A)** Author collaboration network. **(B)** H-index, G-index, and publication output of the top 10 authors by publication output (including ties; 15 authors in total). **(C)** Temporal distribution of publications by core authors.

**Figure 5** shows the author collaboration network, the distribution of core authors, and the temporal characteristics of author output in paraptosis research. Overall, author collaboration in this field was characterized by relatively independent collaborative clusters, with limited direct connections among different clusters. In the author collaboration network (**Figure 5A**), the group represented by Choi Kyeong Sook, Lee Dong Min, Kim In Young, and Seo Min Ji showed the closest connections and formed one of the most prominent collaborative clusters. The author groups including Ahn Kwang Seok, Gandini Valentina, Aoki Shin, Zhao Haobin, and Marzagalli Monica also showed clear clustering patterns. The distribution of core author indicators (**Figure 5B**) showed that Choi Kyeong Sook ranked among the leading authors in publication output, H-index, and G-index. Kessel David and Lee Dong Min also showed relatively high H-index and G-index values, whereas Ahn Kwang Seok had a particularly high G-index, suggesting that these authors had high academic activity and influence in this field. The temporal distribution of author publications (**Figure 5C**) indicated that Gandini Valentina, Marzano Cristina, Pellei Maura, and Santini Carlo entered the field relatively early, whereas Choi Kyeong Sook, Ahn Kwang Seok, Lee Dong Min, Kim In Young, and Seo Min Ji have remained highly active in recent years and constitute a currently active group of core authors. Among them, Choi Kyeong Sook had a long active period and continuously participated in paraptosis research over an extended time span.

### 3.3 Publishing journals, co-cited journals, and dual-map overlay analysis

**Figure 6:**
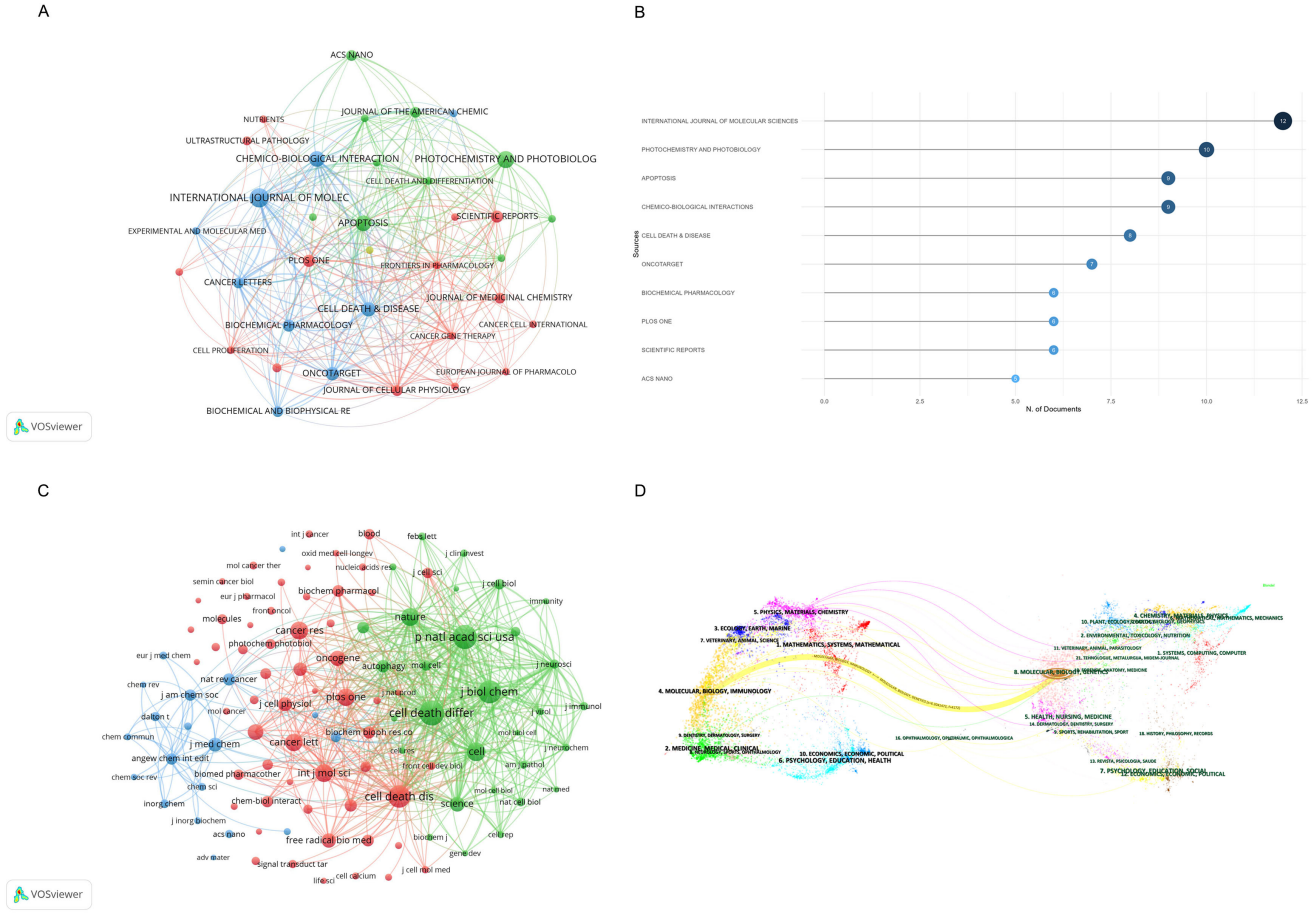
Distribution of publishing journals, journal co-citation network, and journal dual-map overlay. **(A)** Distribution of publishing journals. **(B)** Top 10 journals by publication output. **(C)** Journal co-citation network. **(D)** Journal dual-map overlay.

**Figure 6** shows the distribution of publishing journals, the co-cited journal network, and the journal dual-map overlay in paraptosis research. Overall, the journal distribution demonstrated clear interdisciplinary characteristics. The publishing journal network (**Figure 6A**) showed that paraptosis-related studies were published in journals forming three main clusters, mainly involving cell death and related mechanisms, cancer and molecular mechanisms, and chemical biology and drug research. Cross-links were observed among these clusters, indicating that research outputs in this field were distributed across multiple interconnected journals. Among them, Apoptosis was located near the center of the network and maintained connections with several clusters, suggesting that it played an important bridging role in the publication landscape of paraptosis research. The distribution of highly productive journals (**Figure 6B**) showed that International Journal of Molecular Sciences had the highest publication output (n = 12), followed by Photochemistry and Photobiology (n = 10), Apoptosis (n = 9), and Chemico-Biological Interactions (n = 9). These findings indicate that paraptosis-related studies were mainly concentrated in journals related to cell death mechanisms and interventional research, although the publication advantage of the leading journals was relatively limited. The co-cited journal network (**Figure 6C**) formed three relatively distinct clusters. The green cluster, centered on Proceedings of the National Academy of Sciences of the United States of America, Cell Death & Differentiation, Journal of Biological Chemistry, Nature, and Cell, mainly represented basic life sciences and cell death-related research. The red cluster, with Cell Death & Disease, Cancer Letters, International Journal of Molecular Sciences, Cancer Research, and Oncogene as major nodes, mainly reflected cancer and molecular mechanism-related research. The blue cluster was relatively small and was represented by Journal of Medicinal Chemistry, Angewandte Chemie International Edition, and Journal of the American Chemical Society, reflecting chemical and drug-related research. The journal dual-map overlay (**Figure 6D**) indicated a clear pattern of interdisciplinary knowledge flow in paraptosis research. The major citation path extended from Molecular, Biology, Immunology to Molecular, Biology, Genetics. Medicine, Medical, Clinical and Physics, Materials, Chemistry were also linked to Molecular, Biology, Genetics, indicating that research in this field strongly depends on basic life science knowledge. In addition, Physics, Materials, Chemistry was linked to Health, Nursing, Medicine, and Chemistry, Materials, Physics was linked to Environmental, Toxicology, Nutrition, suggesting that knowledge dissemination in paraptosis research also maintains cross-field connections with medical and chemical research.

### 3.4 Co-cited authors, co-cited references, and intellectual base analysis

#### 3.4.1 Co-cited author and co-cited reference analysis

**Figure 7:**
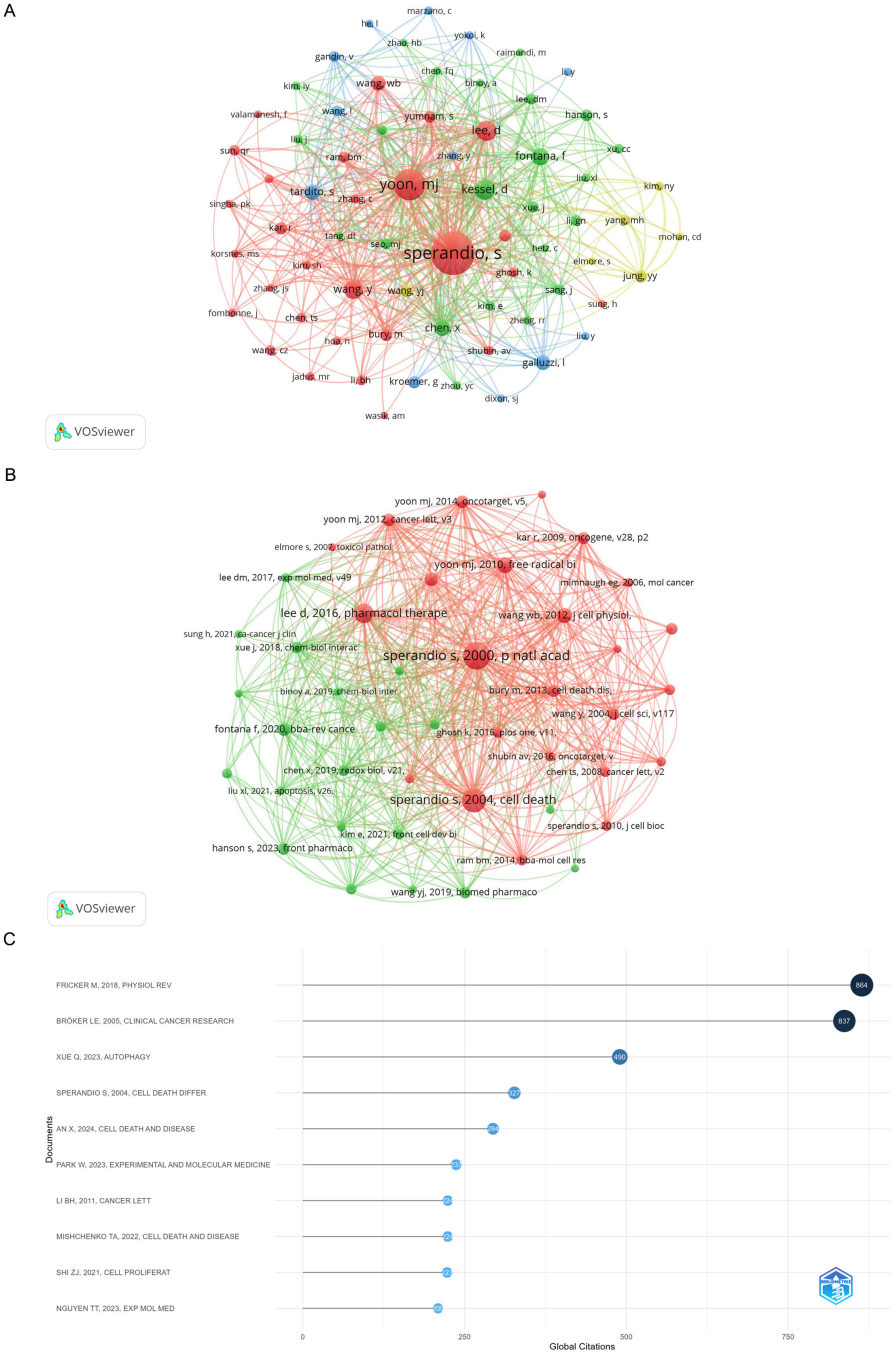
Co-cited authors, co-cited references, and highly cited publications. **(A)** Co-cited author network. **(B)** Co-cited reference network. **(C)** Top 10 highly cited publications.

**Figure 7** shows the co-cited author network, co-cited reference network, and distribution of highly cited publications in paraptosis research. The co-cited author network (**Figure 7A**) showed that co-cited authors in paraptosis research formed interconnected clusters. Sperandio S had the highest co-citation frequency (n = 356), the largest node, and a central position in the network, making this author the most central co-cited author in the field. Yoon MJ (n = 209), Kessel D (n = 101), and Lee D (n = 100) also had high co-citation frequencies, suggesting considerable academic influence in paraptosis research. The co-citation frequencies of authors are shown in **Supplementary Table 2**. The co-cited reference network (**Figure 7B**) revealed the intellectual base of paraptosis research. Sperandio S, 2000, Proc Natl Acad Sci U S A (174 citations) and Sperandio S, 2004, Cell Death Differ (139 citations) were located at the center of the network and were the most representative co-cited references in the field, corresponding to the proposal of the paraptosis concept and early mechanistic investigation, respectively (3, 6). In addition to these foundational studies, Lee D, 2016, Pharmacol Ther (97 citations), Yoon MJ, 2010, Free Radic Biol Med (69 citations), and Wang WB, 2012, J Cell Physiol (59 citations) also formed strong co-citation links and mainly involved paraptosis induction and related mechanistic exploration (13–15). The co-citation frequencies of references are shown in **Supplementary Table 3**. The distribution of highly cited publications (**Figure 7C**) provided a complementary perspective on the impact of individual studies. The globally most cited publications were Fricker M, 2018, Physiol Rev (864 citations) and Bröker LE, 2005, Clin Cancer Res (837 citations), indicating that paraptosis research relies not only on classical literature within the field but also maintains strong links with broader cell death-related research (16, 17). Meanwhile, Sperandio S, 2004, Cell Death Differ remained among the most highly cited publications, further supporting its importance in the knowledge system of paraptosis research.

#### 3.4.2 Reference citation burst analysis

**Figure 8:**
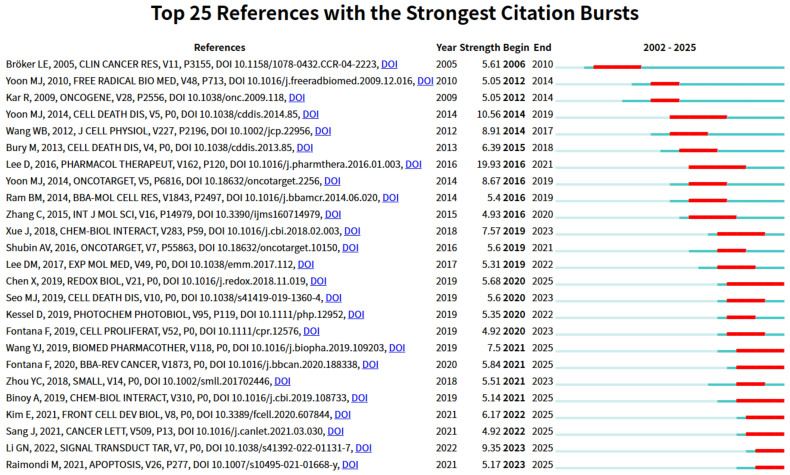
Top 25 cited references with the strongest citation bursts. Citation burst detection was performed with γ = 1.0 and a minimum duration of 2 years.

**Figure 8** shows that reference citation bursts in paraptosis research displayed a clear temporal evolution. Early burst references included Bröker LE, 2005, Clin Cancer Res; Kar R, 2009, Oncogene; and Yoon MJ, 2010, Free Radic Biol Med, indicating that early research mainly focused on the identification and induction of paraptosis-related cell death (14, 17, 18). References with bursts continuing to 2025 included Chen X, 2019, Redox Biol (retracted in 2026; cited here only to objectively present the citation burst result); Wang YJ, 2019, Biomed Pharmacother; Fontana F, 2020, BBA Rev Cancer; Binoy A, 2019, Chem Biol Interact; Kim E, 2021, Front Cell Dev Biol; Sang J, 2021, Cancer Lett; Li GN, 2022, Signal Transduct Target Ther; and Raimondi M, 2021, Apoptosis, suggesting that recent research has continued to focus on molecular mechanisms, regulatory networks, and review-based synthesis related to paraptosis (10, 19–25). Burst strength and burst duration reflect different dimensions of the influence of cited references. A higher burst strength indicates that a reference received rapid and concentrated citation attention within a specific period, whereas a longer burst duration indicates that the reference maintained citation activity over consecutive years. Lee D, 2016, Pharmacol Ther had the highest burst strength (strength = 19.93), followed by Yoon MJ, 2014, Cell Death Dis (strength = 10.56) and Li GN, 2022, Signal Transduct Target Ther (strength = 9.35). Among these references, Yoon MJ, 2014, Cell Death Dis and Lee D, 2016, Pharmacol Ther also showed the longest burst durations. These findings indicate that review-based synthesis, paraptosis induction studies, and mechanistic exploration received concentrated citation attention and maintained sustained influence over multiple years (10, 13, 26).

### 3.5 Keyword analysis: research hotspots, thematic structure, and evolving frontiers

#### 3.5.1 Keyword co-occurrence and clustering analysis

**Figure 9:**
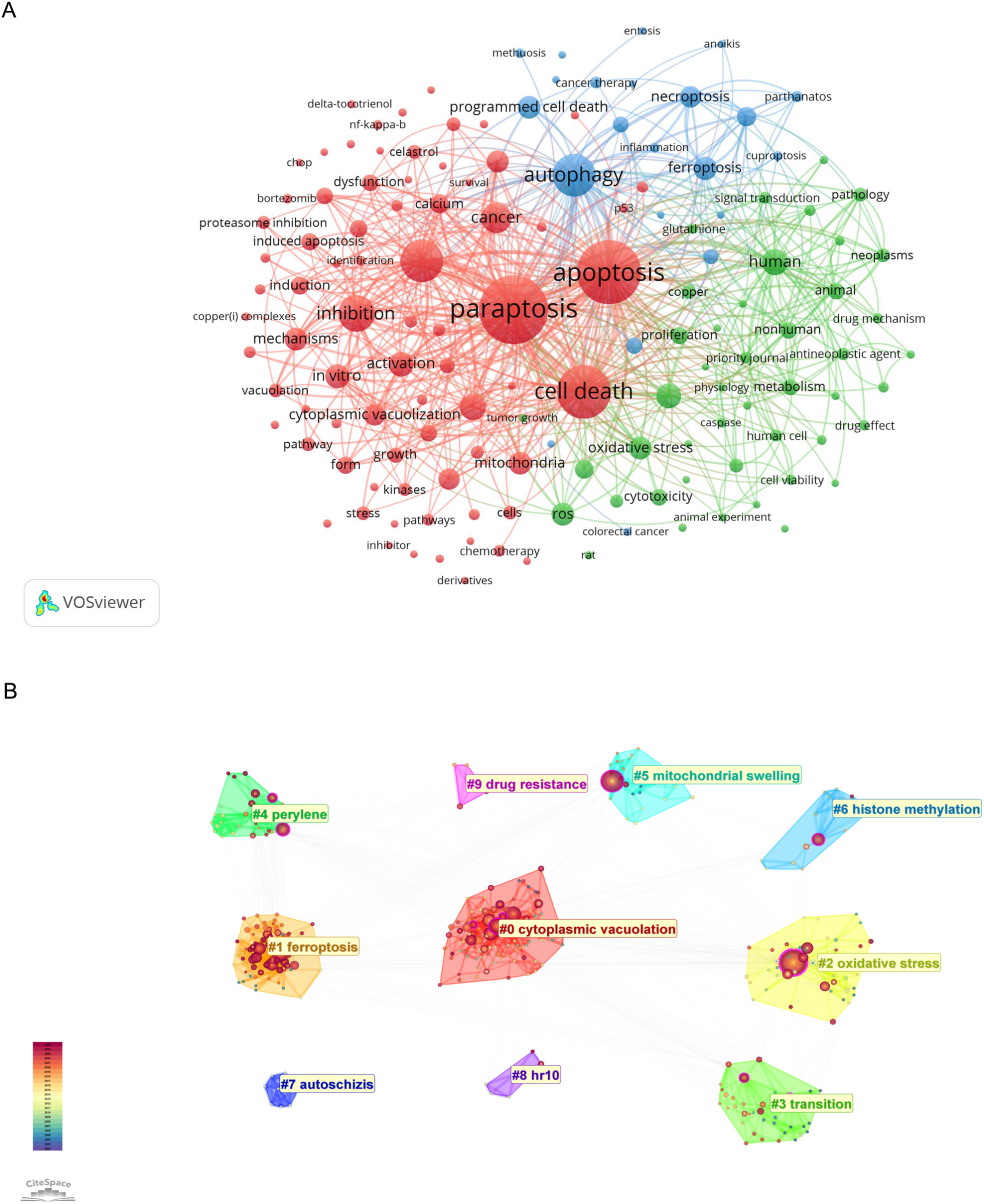
Keyword co-occurrence network and clustering map. **(A)** Keyword co-occurrence network. **(B)** Keyword clustering map.

**Figure 9** presents the keyword co-occurrence network and clustering characteristics of paraptosis research. Overall, the keyword network was centered on paraptosis and formed a relatively clear thematic structure involving cell death types, mechanistic research, and cancer-related topics. The keyword co-occurrence network (**Figure 9A**) showed that paraptosis (220 occurrences) was closely connected with apoptosis (191 occurrences), cell death (141 occurrences), and autophagy (96 occurrences), indicating that paraptosis research was most closely related to cell death-related themes. At the same time, ferroptosis, programmed cell death, necroptosis also maintained strong associations with the core network, suggesting that the relationship and distinction between paraptosis and other forms of cell death are important concerns in the field. Mechanism-related keywords also occupied important positions in the core network, indicating that mechanistic research is a central hotspot in paraptosis research. Endoplasmic reticulum stress, endoplasmic reticulum, mitochondria, ROS, and oxidative stress were strongly linked with paraptosis, suggesting that ER stress, organelle alterations involving the ER and mitochondria, and oxidative stress are currently prominent mechanistic directions. In addition, high-frequency keywords such as cancer, cancer cells, and anticancer were also closely connected with paraptosis, indicating that cancer-related research is an important application context. High-frequency keywords are shown in **Supplementary Table 4**.

Keyword clustering (**Figure 9B**) showed that paraptosis research has formed relatively clear thematic modules. The largest cluster, #0 cytoplasmic vacuolation, corresponds to the most representative morphological feature of paraptosis. Clusters #2 oxidative stress and #5 mitochondrial swelling reflect that oxidative stress and mitochondrial alterations are sustained mechanistic themes in this field. Cluster #1 ferroptosis and cluster #7 autoschizis suggest that comparative research between paraptosis and other non-canonical forms of cell death occupies an important position. Cluster #4 perylene, cluster #6 histone methylation, and cluster #9 drug resistance indicate that the field is extending toward specific inducers, epigenetic regulation, and resistance-related research. By comparison, cluster #3 transition and cluster #8 hr10 were less representative of their thematic content and did not directly indicate clearly concentrated research themes, suggesting a certain degree of thematic overlap within these clusters. Overall, keyword clustering indicated that paraptosis research has developed a multi-thematic structure centered on typical phenotypes, mechanistic investigation, comparison among cell death modes, and specific induction contexts.

#### 3.5.2 Keyword-based thematic evolution and thematic quadrant analysis

**Figure 10:**
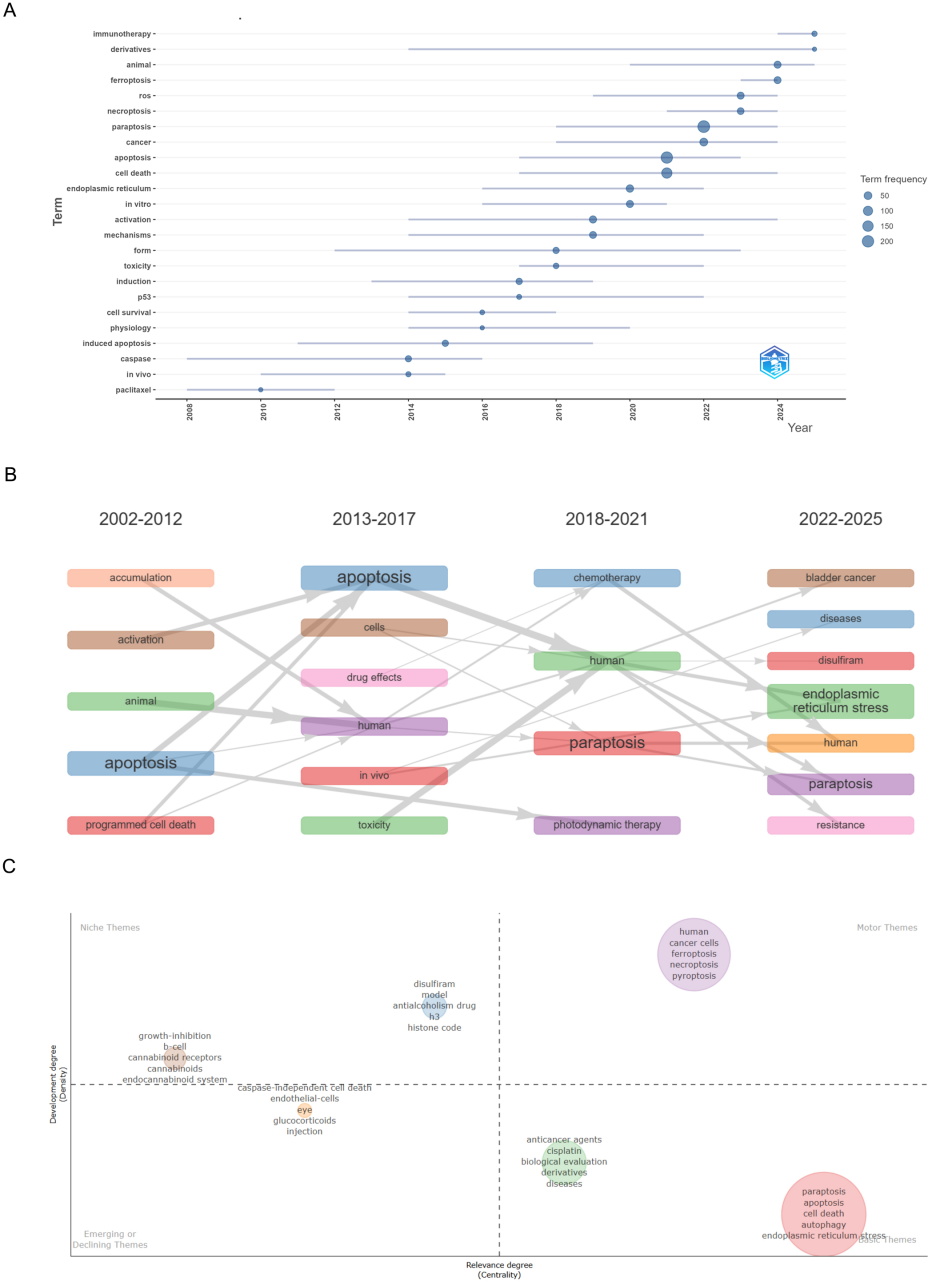
Temporal distribution of themes, thematic evolution, and thematic quadrant map. **(A)** Temporal distribution of themes. **(B)** Thematic evolution map. **(C)** Thematic quadrant map.

**Figure 10** presents the dynamic changes in paraptosis research hotspots from three perspectives: temporal distribution of themes, stage-based evolution, and thematic structure. **Figure 10A** shows that themes differed in time of emergence, duration, and level of attention. Early themes included paclitaxel, *in vivo*, and caspase, suggesting that early studies were closely related to drug induction, *in vivo* experiments, and classical cell death topics. Themes such as p53, form, mechanisms, and activation persisted over relatively long periods, indicating sustained attention to the forms, activation processes, and regulatory mechanisms of paraptosis. Derivatives have continued to appear since 2014, suggesting persistent attention to derivative-related research. More recently emerging themes such as ferroptosis and immunotherapy indicate that current research has extended to other non-canonical forms of cell death and immunotherapy-related directions. In addition, large nodes for apoptosis, cell death, cancer, and paraptosis reflect high attention within the research network.

**Figure 10B** reveals the evolutionary paths of research themes across different stages. From 2002 to 2012, early research mainly focused on comparative identification and experimental observations within the broader context of cell death. From 2013 to 2017, research themes gradually expanded toward drug effects and experimental validation while continuing cell death-related research. From 2018 to 2021, paraptosis emerged as an independent and relatively large theme, and chemotherapy and photodynamic therapy also appeared, indicating that paraptosis research became more independent while integrating with applied strategies such as chemotherapy and photodynamic therapy. From 2022 to 2025, research themes further extended toward mechanisms, drugs, and disease applications. Among these, the endoplasmic reticulum stress module was particularly prominent, indicating that it has become an important mechanistic theme in recent paraptosis research.

**Figure 10C** presents the current status and maturity of paraptosis research themes from the dimensions of centrality and density. The upper-right quadrant represents motor themes. The themes in this quadrant were relatively large and positioned high in the map, mainly involving cancer-related research and the connections between paraptosis and other forms of cell death, including ferroptosis, necroptosis, and pyroptosis, indicating that these directions are major drivers of field expansion. The lower-right quadrant represents basic themes. One larger thematic cluster, centered on paraptosis, apoptosis, cell death, autophagy, and endoplasmic reticulum stress, indicates that the relationships between paraptosis, other forms of cell death, and ER stress constitute a foundational research domain with high centrality but relatively limited internal development. Another thematic cluster was related to anticancer agents, cisplatin, derivatives, and diseases, suggesting that research on anticancer drugs and their derivatives is closely linked to the main body of the field. The upper-left quadrant represents niche themes, mainly involving disulfiram, histone code, and cannabinoid-related themes, indicating specialized research directions related to drugs and cannabinoids, although their connections with the overall research backbone remain relatively limited. The lower-left quadrant represents emerging or declining themes, suggesting that some relatively marginal topics still exist in paraptosis research.

## 4 Discussion

### 4.1 Overall research profile

Based on paraptosis-related publications indexed in WoSCC, PubMed, and Scopus, this study systematically analyzed the current development status of the field. The results showed that publication output on paraptosis exhibited an overall fluctuating upward trend. After the concept was proposed, the field experienced a prolonged period of low publication output, followed by more sustained growth in recent years. It should be noted that the concept of paraptosis was first proposed in 2000, whereas the annual distribution of publications included in this study began in 2002. This suggests that early related studies did not consistently use the term “paraptosis.” Therefore, the developmental trajectory presented more accurately reflects the evolution of research centered on “paraptosis” as the core topic term. Compared with more established forms of cell death, such as apoptosis and ferroptosis, the overall literature volume of paraptosis remains relatively limited, indicating that the research system of this field is still under development. Nevertheless, sustained publication growth in recent years, continuous differentiation of hotspots, and gradual expansion of application contexts suggest that paraptosis research is moving from basic recognition toward deeper mechanistic and applied investigation.

### 4.2 Research strength and collaboration patterns

Analyses of countries, institutions, and authors showed that paraptosis research has formed several relatively active research forces, although the overall collaboration network remains limited in scale. International collaboration was mainly concentrated among China, the United States, and South Korea, with the China-United States collaboration being the closest. Institutional collaboration formed several local collaborative groups, and cross-links existed among different institutions. Although highly productive institutions such as Ajou University and Kyung Hee University were relatively active in the network, a dominant advantage among leading institutions has not yet fully emerged. Author collaboration was characterized by relatively stable intra-team cooperation, whereas cross-team communication was limited, suggesting that this field is still driven to some extent by a small number of core research groups.

Journal analysis indicated that paraptosis research has clear interdisciplinary characteristics. Relevant studies were mainly published in journals related to cell death mechanisms, cancer molecular mechanisms, and chemical biology or drug research. The intellectual base of the field is centered on basic life sciences and maintains stable cross-disciplinary connections with medicine and chemistry. Overall, paraptosis research currently shows a developmental pattern in which a small number of active countries, institutions, and author teams play leading roles while the field continues to absorb interdisciplinary knowledge and emerging research forces.

### 4.3 Keyword-based research hotspot analysis

Keyword-related analyses provide a focused perspective for understanding the main hotspots in paraptosis research. Based on keyword co-occurrence, clustering, and thematic analyses, this section summarizes the current research hotspots in the field. The largest keyword cluster, cytoplasmic vacuolation, corresponds to the most representative morphological feature of paraptosis, suggesting that phenotypic identification constitutes an important foundation of this field. The keyword co-occurrence network showed that paraptosis had clear co-occurrence links with other forms of cell death, such as apoptosis, autophagy, and ferroptosis. In the thematic quadrant map, apoptosis and autophagy were important components of basic themes, whereas ferroptosis, pyroptosis, and necroptosis appeared in the motor-theme quadrant. Among these, ferroptosis was also represented in keyword clustering and thematic evolution. These findings indicate that paraptosis has not been discussed in isolation but has progressed within a broader research context involving multiple forms of cell death, and that its positioning and recognition may continue to be shaped through comparison with, and distinction from, other cell death modalities. Meanwhile, keywords such as endoplasmic reticulum stress, mitochondria, ROS, and mechanisms occupied important positions in the co-occurrence network. Together with the clustering results for oxidative stress and mitochondrial swelling, these findings suggest that mechanistic investigation constitutes a central focus of paraptosis research. Among these mechanistic directions, ER stress, mitochondrial alterations, and ROS-related processes are particularly representative. In addition, high-frequency keywords such as cancer, cancer cells, and anticancer were clearly associated with paraptosis, and the appearance of themes such as anticancer agents, cisplatin, resistance, chemotherapy, photodynamic therapy, and immunotherapy collectively indicates that cancer-related research represents a prominent disease context and application-oriented direction in the paraptosis field. Overall, the keyword-related results suggest that current paraptosis research hotspots can be summarized into four major aspects: phenotypic identification, comparison with other forms of cell death, exploration of potential mechanisms, and cancer-related research.

### 4.4 Literature synthesis of major hotspots in paraptosis research

**Figure 11:**
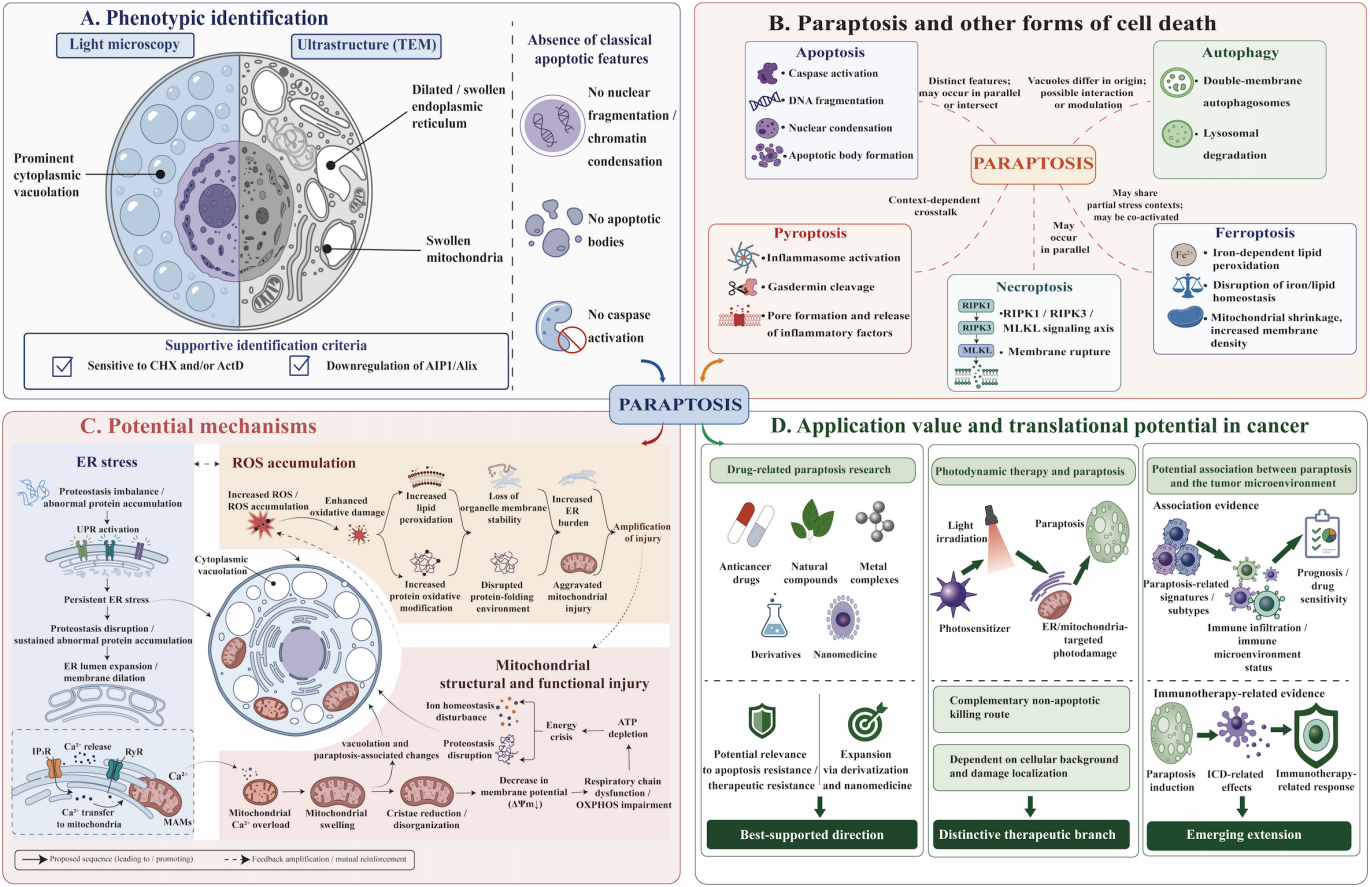
Schematic overview of major research hotspots in paraptosis identified from bibliometric analysis. TEM, transmission electron microscopy; CHX, cycloheximide; ActD, actinomycin D; AIP1/Alix, ALG-2-interacting protein X/apoptosis-linked gene 2-interacting protein X; ER, endoplasmic reticulum; UPR, unfolded protein response; IP3R, inositol 1, 4, 5-trisphosphate receptor; RyR, ryanodine receptor; MAMs, mitochondria-associated membranes; ROS, reactive oxygen species; OXPHOS, oxidative phosphorylation; ICD, immunogenic cell death.

Based on the summary of research hotspots above, this section synthesizes existing literature to further discuss the major research hotspots in paraptosis, with the aim of clarifying the current understanding and potential value of these topics. **Figure 11** provides a schematic overview of the overall framework and major contents of this literature synthesis.

#### 4.4.1 Phenotypic identification of paraptosis

When Sperandio et al. first proposed the concept of paraptosis (3), they described its core morphological feature as marked cytoplasmic vacuolation, with vacuoles mainly derived from swollen and expanded ER and/or mitochondria. Paraptosis also lacks the nuclear condensation, apoptotic body formation, chromatin condensation, and caspase dependence commonly observed in classical apoptosis. In addition, paraptosis depends on protein synthesis and can be blocked by the transcription inhibitor actinomycin D (ActD) and the translation inhibitor cycloheximide (CHX). Subsequent studies further identified AIP1/Alix as a negative regulator of paraptosis (6), and changes in its expression are often used as molecular evidence of paraptosis. Therefore, paraptosis identification does not rely on a single marker; rather, it is usually based on multidimensional evidence, including cytoplasmic vacuolation, absence of classical apoptotic features, downregulation of AIP1/Alix, and sensitivity to protein synthesis inhibitors. Recent experimental studies have generally followed this phenotypic identification framework across different model systems. For example, Lin et al. used triple-negative breast cancer cells and identified a paraptosis phenotype based on cytoplasmic vacuolation, mitochondrial swelling, and negative detection of caspase/PARP cleavage (27). Li et al. used drug-resistant ovarian cancer cells, and Wei et al. used hepatocellular carcinoma cells to support paraptosis through cytoplasmic vacuolation, ER or mitochondrial swelling, insensitivity to apoptosis inhibitors, and sensitivity to CHX (10, 28). Hou et al. further confirmed paraptosis in osteoarthritis-related chondrocytes by integrating the above evidence with reduced Alix protein expression (29). Taken together, these studies support the view that phenotypic identification of paraptosis requires comprehensive judgment, with cytoplasmic vacuolation serving as the core starting point.

#### 4.4.2 Connections and distinctions between paraptosis and other forms of cell death

This section discusses the connections and distinctions between paraptosis and several representative forms of cell death, with their main similarities and differences summarized in **Supplementary Table 5**.

##### 4.4.2.1 Paraptosis and apoptosis

Distinguishing paraptosis from apoptosis is of fundamental importance. Paraptosis gained independent status through its morphological and biochemical differences from apoptosis. Apoptosis is typically characterized by activation of the caspase family, DNA fragmentation, nuclear condensation, and formation of apoptotic bodies (30), whereas paraptosis lacks these classical apoptotic features and is primarily manifested by cytoplasmic vacuolation and ER and/or mitochondrial expansion (3). Although both belong to PCD, their execution programs and identification criteria differ. The high frequency of apoptosis in the keyword network reflects that paraptosis research has long developed within a theoretical context of differentiation from apoptosis, which is important for the phenotypic identification of paraptosis. Related studies have suggested that paraptosis should be regarded as a death program parallel to apoptosis and that, under certain stress conditions, it may occur in parallel with or intersect with apoptosis (2).

##### 4.4.2.2 Paraptosis and autophagy

The relationship between paraptosis and autophagy is more complex. Both can be accompanied by vacuole-like structures and may therefore be confused morphologically, but their biological implications and regulatory nature differ. Autophagy is centered on double-membrane autophagosomes and lysosomal degradation pathways, and its vacuoles are mainly related to the formation of autophagosomes and autolysosomes (31). In contrast, paraptosis-associated vacuoles are mainly derived from ER and/or mitochondrial expansion and usually do not depend on the classical autophagic degradation program. Kar et al. found that paraptosis-like vacuolation could be accompanied by LC3 processing and changes in autophagy-related proteins, suggesting that autophagy may play an accompanying or regulatory role in some studies of paraptosis (18). Recent reviews have also indicated possible interactions between paraptosis and autophagy (32). In the context of the present study, the high-frequency co-occurrence of autophagy suggests that vacuolation is not specific to paraptosis. Distinguishing vacuolar changes among different forms of cell death therefore remains an important issue in paraptosis research.

##### 4.4.2.3 Paraptosis and ferroptosis

Ferroptosis is a form of PCD centered on iron-dependent lipid peroxidation (33). Both ferroptosis and paraptosis are associated with intracellular homeostatic imbalance, but their dominant mechanisms differ. Ferroptosis mainly involves disruption of iron metabolism and lipid metabolism homeostasis (34), whereas paraptosis is more closely related to proteostasis imbalance (5). The two also differ in organelle morphology: ferroptosis is characterized by reduced mitochondrial volume, increased membrane density, and decreased cristae, whereas paraptosis is characterized by mitochondrial and/or ER swelling (35). Studies have shown that these two death modes share certain similarities in ROS changes and MAPK pathway involvement. In paraptosis, ROS promotes cytoplasmic vacuolation through ER stress and mitochondrial dysfunction. In ferroptosis, ROS drives iron-mediated Fenton reactions, catalyzes lipid peroxidation chain reactions, and ultimately disrupts cell membrane integrity (36). Yang et al. found that micheliolide can simultaneously activate paraptosis and ferroptosis by regulating the MAPK pathway (37). Wang et al. found that iron chelators may induce paraptosis by triggering sustained ferritinophagy (38), suggesting that iron metabolism-related interventions may be linked to paraptosis. In addition, both paraptosis and ferroptosis are considered potential alternative death routes for overcoming apoptosis evasion in cancer therapy (39, 40). Therefore, ferroptosis and paraptosis share some regulatory features and may be induced simultaneously, although the specific mechanistic relationship and functional interaction between them remain unclear. The high frequency of ferroptosis in this study mainly suggests that paraptosis may share certain regulatory mechanisms and potential application value with ferroptosis.

##### 4.4.2.4 Paraptosis and other non-canonical forms of cell death

Necroptosis and pyroptosis also appeared in the keyword network. Necroptosis is a form of programmed necrosis mediated by the RIPK1/RIPK3/MLKL signaling axis and is characterized by cell membrane rupture (41). Pyroptosis is an inflammatory form of cell death that depends on inflammasome activation and Gasdermin cleavage and is characterized by pore formation and inflammatory factor release (42). Necroptosis and pyroptosis differ from paraptosis in both molecular mechanism and morphology. Research linking them with paraptosis remains limited, and current evidence more strongly supports the possibility that they may occur in parallel with, or synergistically with, paraptosis under specific conditions, while mechanistic connections remain insufficiently demonstrated. Dewan et al. found that the gold(I)-NHC complex AuL4 induced both necroptosis and paraptosis in lung cancer cells, suggesting that the two can co-occur as parallel non-canonical cell death modes and may have potential value when apoptosis is limited in cancer cells (43). Chen et al. reported that tumor microenvironment-responsive zinc-nickel bimetallic hydroxide nanosheets could induce a paraptosis-pyroptosis positive feedback loop, in which nickel ions primarily triggered paraptosis and zinc ions mainly promoted pyroptosis; their synergistic action enhanced antitumor immunotherapy (44). The appearance of necroptosis and pyroptosis suggests that paraptosis research is increasingly focusing on relationships with other non-canonical cell death modes and on its functional positioning. However, their specific mechanistic links and disease-related application value still require further investigation.

#### 4.4.3 Potential mechanisms of paraptosis

##### 4.4.3.1 Endoplasmic reticulum stress

Both keyword co-occurrence and thematic evolution suggested that ER stress is one of the most representative mechanisms identified in this study. Research indicates that proteostasis imbalance is closely associated with paraptosis (4). The ER is responsible for protein folding, modification, and transport. When misfolded or unfolded proteins accumulate persistently, or when the capacity of the proteasome and ER-associated degradation system declines, cells initiate the unfolded protein response (UPR) to restore homeostasis. During the initial phase, UPR activation is compensatory. However, if disruption of protein folding persists, prolonged UPR activation fails to restore homeostasis, and ER function shifts from adaptive regulation to sustained stress, leading to intracellular proteostasis disruption and continued accumulation of abnormal proteins (45). Persistent proteostasis disruption can cause continuous expansion of the ER lumen and swelling of membranous structures, eventually forming abundant cytoplasmic vacuoles. Thus, ER stress provides a structural basis for the formation of the vacuolated phenotype in paraptosis. ER stress can also disrupt intracellular Ca^2+^ homeostasis. The ER is the major intracellular Ca^2+^ store. When ER stress persists, Ca^2+^ can be released into the cytoplasm through inositol 1, 4, 5-trisphosphate receptors (IP3Rs), ryanodine receptors (RyRs), and mitochondria-associated ER membrane contact sites and can then be taken up by adjacent mitochondria. The resulting mitochondrial Ca^2+^ overload may cause mitochondrial swelling, decreased membrane potential, impaired respiratory chain function, and insufficient ATP supply, leading to structural and functional mitochondrial damage (23). In addition, sustained ER stress promotes ROS elevation, and ROS accumulation can, in turn, damage the protein-folding environment and aggravate oxidation of membrane lipids and proteins, further amplifying the injury cycle between the ER and mitochondria (7). Xu et al. showed that metal nanoparticles can induce persistent ER stress by disrupting ER membrane integrity, resulting in massive Ca^2+^ entry into mitochondria and increased ROS generation. ROS then further damages the ER membrane, causing additional ER swelling and accumulation of misfolded proteins, ultimately inducing paraptosis (46). Overall, existing studies support an important role of ER stress in paraptosis and suggest that it may be a key link connecting proteostasis imbalance, cytoplasmic vacuolation, and organelle dysfunction.

##### 4.4.3.2 Mitochondrial structural and functional injury

Mitochondrial alterations represent another mechanism that has received sustained attention in paraptosis research. During paraptosis, mitochondria frequently exhibit swelling, reduced or disorganized cristae, and membranous expansion; these changes are also one source of paraptosis-related vacuolar structures (47). Multiple studies have indicated that changes in mitochondrial membrane potential, abnormal energy metabolism, and mitochondrial dysfunction appear together with the paraptotic phenotype, suggesting that mitochondrial abnormalities may be important contributors to paraptosis (47, 48).

Mitochondrial dysfunction can place cells in an energy crisis and may affect the progression of paraptosis. Raimondi et al. detected downregulation of oxidative phosphorylation complex I, reduced oxygen consumption, decreased mitochondrial membrane potential, and reduced ATP production during delta-tocotrienol-induced paraptosis in melanoma cells (25). He et al. also found in experiments with mitochondria-targeted iridium complexes that paraptosis was accompanied by rapid loss of mitochondrial membrane potential, ATP depletion, and respiratory inhibition, followed by mitochondrial collapse and mitochondria-derived cytoplasmic vacuolation (48). These results suggest that paraptosis is often accompanied by pronounced impairment of mitochondrial energy metabolism. Mitochondrial damage may also be accompanied by imbalance in transmembrane ion transport, among which abnormal ER-mitochondrial Ca^2+^ transport is a repeatedly observed mechanistic event in paraptosis research. Following mitochondrial Ca^2+^ overload, osmotic balance in mitochondria may be disrupted, leading to increased water influx into the matrix and subsequent mitochondrial swelling and cristae disorganization (25). Meanwhile, cristae damage further weakens the spatial organization of the electron transport chain and the efficiency of oxidative phosphorylation, causing decreased membrane potential and impaired ATP generation (26). As mitochondrial energy supply declines, the ability of cells to maintain ion gradients, volume homeostasis, and proteostasis also decreases, thereby promoting organelle expansion and continued aggravation of cytoplasmic vacuolation (2). Some studies have directly linked paraptosis with mitochondrial Ca^2+^ overload and mitochondrial dysfunction (47, 49), further emphasizing the importance of mitochondria and their functional imbalance in paraptosis. Imbalance of mitochondrial channels themselves may also contribute to ion homeostasis disruption and affect paraptosis. Bury et al. showed that Ophiobolin A can reduce BKCa channel activity, disrupt intracellular ion homeostasis, induce swelling of mitochondria and ER, and trigger paraptosis (50).

In mitochondria-associated paraptosis, mitochondrial dysfunction is often accompanied by increased mitochondria-derived ROS production and is linked to activation of the MAPK pathway and promotion of cytoplasmic vacuolation, allowing early mitochondrial damage to develop into a typical paraptotic phenotype (25). Mitochondrial dysfunction and proteostasis disruption may also influence each other. Loss of mitochondrial membrane potential, respiratory inhibition, and ATP depletion can affect cellular protein quality-control processes, particularly ubiquitinated protein degradation mediated by the 26S proteasome (51). At the same time, enhanced oxidative stress can impair the stability and activity of the 26S proteasome and further aggravate protein damage and abnormal protein accumulation (52, 53). However, the specific coupling of this relationship in paraptosis remains to be clarified. Overall, mitochondrial structural destruction and functional impairment are important mechanistic components of paraptosis. Through energy imbalance, ion homeostasis disruption, and increased ROS generation, they promote organelle expansion and drive the paraptotic process.

##### 4.4.3.3 ROS accumulation

ROS is another recurrent theme in mechanistic studies of paraptosis. Existing studies show that paraptosis is often accompanied by marked ROS elevation. Abnormal ROS accumulation may occur early as a stress signal involved in the paraptotic process, or it may occur in parallel with ER stress and mitochondrial damage as part of injury amplification (54–56). Elevated ROS can continuously aggravate intracellular oxidative damage, manifested as enhanced membrane lipid peroxidation and increased oxidative modification of proteins. These changes further disrupt the stability of organelle membrane systems and the protein-folding environment, promoting abnormal protein accumulation (54). Such alterations further increase ER burden and aggravate damage to mitochondrial membrane lipids and protein complexes, making ER stress and mitochondrial dysfunction more difficult to resolve (56). As oxidative damage continues to intensify, organelle expansion and cytoplasmic vacuolation are more likely to progress from reversible stress to irreversible cell death (14). Some studies have found that inhibition of ROS generation significantly attenuates paraptosis-like death (57, 58), supporting the involvement of ROS accumulation in amplification of cellular injury and progression of cell death. ROS accumulation is also closely linked to mitochondrial damage. Elevated ROS can further aggravate mitochondrial structural and functional damage, whereas damaged mitochondria can promote sustained ROS elevation (47, 56), forming a self-amplifying injury loop that drives paraptosis.

In summary, ER stress, mitochondrial damage, and ROS accumulation are important mechanisms in paraptosis. They are intricately interconnected and, together with proteostasis imbalance, Ca^2+^ homeostasis disruption, energy metabolism impairment, and oxidative damage, jointly promote the formation and aggravation of paraptosis-associated phenotypes.

#### 4.4.4 Application value and translational potential of paraptosis in cancer

##### 4.4.4.1 Drug-related paraptosis research

The thematic temporal distribution and thematic evolution results showed that paraptosis research was early linked to paclitaxel, drug effects, and toxicity, and later extended to chemotherapy, anticancer agents, cisplatin, derivatives, disulfiram, and resistance. These findings indicate that drug intervention is one of the stable directions for paraptosis research in cancer. Drug-related paraptosis research is mainly reflected in two aspects. First, paraptosis may serve as a complementary killing strategy to overcome apoptosis resistance and therapeutic resistance. The copper complex and its derivative HydroCuP induced paraptosis in colorectal cancer and showed tumor-suppressive activity in oxaliplatin-sensitive and oxaliplatin-resistant xenograft tumors (59, 60). Elaiophylin preferentially killed ovarian cancer cells resistant to platinum agents, taxanes, and poly(ADP-ribose) polymerase (PARP) inhibitors and overcame therapeutic tolerance in multiple resistant xenograft models by inducing paraptosis (10). A disulfiram-loaded Ca^2+^/Cu^2+^ dual-ion nanoplatform was designed to overcome apoptosis evasion in tumor cells and enhanced breast cancer killing sensitivity by simultaneously inducing paraptosis and apoptosis (61). Together, these studies support the value of paraptosis as a complementary killing mechanism in the context of apoptosis resistance or therapeutic resistance. Second, drug-related paraptosis research has expanded from general compound screening to specific inducers, derivatives, and delivery systems. Dimethoxycurcumin showed stronger paraptosis-inducing effects than curcumin and was accompanied by more prominent proteasome inhibition and ER expansion, suggesting that structural modification of the same parent compound may alter the strength of paraptosis induction (62). Oxidized disulfiram derivatives can induce paraptosis-like death in breast cancer cells (63), suggesting that drug derivatization may also be used as a paraptosis-inducing strategy. At the level of delivery systems, Ca^2+^/Cu^2+^ dual-ion nanoplatforms strengthened paraptosis-related responses through ion co-delivery (61), indicating that the delivery mode itself may be a research direction for regulating paraptosis. Studies on cyclometalated iridium complex-cationic peptide hybrids and inhibition of the mitochondrial Na^+^/Ca^2+^ exchanger also indicate that paraptosis induction has begun to integrate with subcellular targeting and ion-flux regulation (64, 65). It should be noted that different drug interventions vary in organelle targets, stress axes, and concurrent cell death modes. Therefore, whether paraptosis is the major killing mode or coexists with other death modes requires evaluation according to the specific study context.

##### 4.4.4.2 Photodynamic therapy and paraptosis

The thematic evolution results showed that photodynamic therapy appeared as a relatively independent theme during 2018-2021, and journal analysis showed that Photochemistry and Photobiology ranked among the highly productive journals. These findings indicate that photodynamic therapy (PDT) is a relatively distinctive therapeutic research direction in the paraptosis field. Existing studies suggest that the link between PDT and paraptosis is mainly based on differences in subcellular damage localization. Kessel et al. reported that PDT can induce paraptosis in multiple cancer cells through ER photodamage and may still exert cytotoxic effects in cells with impaired or apoptosis-insensitive responses (66, 67). Related studies further suggested that paraptosis induced by ER/mitochondrial photodamage shows clear cell-context dependence, indicating that its occurrence depends not only on the subcellular localization of photosensitizer-induced damage, but also on the intrinsic death-response state of the cell (68). A comparative study by Lange et al. on mTHPC-mediated PDT showed that different tumor cells can exhibit different death pathways under the same PDT conditions, with paraptosis constituting an important component (69). This suggests that paraptosis is related to cell background and damage-output patterns. Cho et al. also observed in head and neck squamous cell carcinoma models that ER/mitochondria-targeted PDT can first induce paraptosis and then proceed to other programmed death processes (70). These studies indicate that, when apoptotic responses are limited, ER- and/or mitochondria-targeted photodamage can still induce paraptosis, and paraptosis may therefore constitute a complementary pathway by which PDT maintains its cytotoxic effect (66, 68). Compared with drug treatment, PDT has the advantage of more clearly defining damage sites, making it more suitable for observing the relationship between subcellular damage localization and cell death mode. However, current research on PDT and paraptosis is much less extensive than drug-related research and remains concentrated in a small number of research teams and specific photosensitizer systems. Therefore, its generalizability across different photosensitizers, subcellular targeting approaches, and tumor models requires further validation.

##### 4.4.4.3 Potential links between paraptosis and the tumor microenvironment

The appearance of immunotherapy in thematic temporal evolution suggests that paraptosis research has begun to extend toward tumor immunity-related directions. Recent studies also indicate that paraptosis may be linked to the tumor microenvironment (TME) (4). In breast cancer-related research, paraptosis-related genes were associated with immune cell infiltration, immune microenvironmental status, and patient prognosis, suggesting that paraptosis-related molecular features may be useful for evaluating tumor immune status and prognostic risk (71). Studies in gastric cancer similarly showed that paraptosis-related subtypes and risk signatures were associated with immune infiltration, the tumor immune microenvironment, and drug sensitivity, indicating that paraptosis-related molecular patterns can reflect different immune states and therapeutic response backgrounds (72). In the context of immunotherapy, Zheng et al. suggested that the paraptosis inducer CMN can inhibit indoleamine 2, 3-dioxygenase activity, promote ICD, and enhance immunotherapeutic efficacy (11). Liao et al. found that cyclometalated iridium(III) complexes can induce paraptosis and promote ICD in HepG2 cells, enhance the antitumor activity of effector T cells, promote dendritic cell maturation, inhibit the activity of immunosuppressive cells, and thereby strengthen antitumor immune responses (73). At present, research on paraptosis and the TME mainly focuses on immune cell infiltration and immunotherapy-related exploration. Current evidence supports a potential connection between paraptosis and the TME, but the specific modes of action and applicable boundaries of this relationship remain to be clarified.

Overall, drug-induced paraptosis currently represents the major cancer-related direction with the strongest evidence base for paraptosis research in cancer. PDT constitutes a distinctive research pathway, whereas TME-related studies constitute a recent extension of the field.

### 4.5 Limitations

This study systematically summarized the developmental history, overall landscape, and research hotspots of paraptosis through bibliometric methods, but several limitations should be acknowledged. First, this study used “paraptosis” as the search term and required the term to appear explicitly in the title, abstract, or keywords. This strategy improved retrieval specificity, but it may also have excluded relevant studies conducted before “paraptosis” became an established formal term, potentially affecting the early publication distribution. In addition, database selection and language restriction may have led to the omission of some relevant studies, particularly those not indexed in the selected databases or not published in English. Second, the overall size of the paraptosis field remains relatively limited, and network structure and thematic clustering may be susceptible to the influence of core publications and high-frequency keywords. Recently published literature is also affected by citation lag. Although some emerging themes have appeared in keyword temporal evolution and burst analyses, their subsequent influence and stability require further observation. Furthermore, the criteria for identifying paraptosis have not yet been fully unified, which may affect the stability of retrieval, clustering, and thematic identification results.

## 5 Conclusion

In recent years, attention to paraptosis has continued to increase. The field has gradually progressed from early exploration centered on identification of death phenomena and induction observations toward mechanistic elucidation and disease-related applications. Current research hotspots mainly include phenotypic identification, relationships with other forms of cell death, molecular mechanisms, and cancer-related exploration. ER stress, mitochondrial alterations, and ROS accumulation are important mechanistic directions in this field. In cancer research, the potential link between paraptosis and the TME may become an important entry point for expanding antitumor strategies. In the future, strengthening international collaboration and cross-team communication, further clarifying the key regulatory networks and disease-related functional significance of paraptosis, and promoting high-quality applied and translational research may advance this field. This study systematically summarizes research hotspots and developmental trends in the paraptosis field and may provide a reference for subsequent mechanistic research and disease-related applications.

## Data Availability

The original contributions presented in the study are included in the article/**Supplementary Material**. Further inquiries can be directed to the corresponding author.
