# Supplementary material for: Global research landscape and hotspots of paraptosis: a multi-database bibliometric analysis with a focused literature review
Source: Front Oncol. 2026 Jul 21;16:1864713. doi: 10.3389/fonc.2026.1864713 (PMC13433162; doi:10.3389/fonc.2026.1864713)
Supplement: Supplementary file 1 [file Table1.docx]

Supplementary Material

**1 Supplementary Data**

**1.1 Supplementary Tables**

**Table S1.** Top 10 countries by publication volume

| Rank | Country | Documents | Citations |
| --- | --- | --- | --- |
| 1 | China | 155 | 4939 |
| 2 | USA | 66 | 3739 |
| 3 | South Korea | 46 | 1805 |
| 4 | India | 30 | 636 |
| 5 | Italy | 22 | 1550 |
| 6 | Japan | 14 | 270 |
| 7 | France | 12 | 500 |
| 8 | Germany | 12 | 465 |
| 9 | United Kingdom | 9 | 1232 |
| 10 | South Africa | 8 | 215 |
|  | Turkey | 8 | 37 |

**Table S2.** Top 10 co-cited authors

| Rank | Author | Citations | Total link strength |
| --- | --- | --- | --- |
| 1 | Sperandio, S | 356 | 3740 |
| 2 | Yoon, MJ | 209 | 3347 |
| 3 | Kessel, D | 101 | 1432 |
| 4 | Lee, D | 100 | 1257 |
| 5 | Wang, Y | 86 | 1529 |
| 6 | Fontana, F | 77 | 1319 |
| 7 | Chen, X | 68 | 1483 |
| 8 | Tardito, S | 64 | 843 |
| 9 | Wang, WB | 61 | 871 |
| 10 | Galluzzi, L | 60 | 817 |

**Table S3.** Top 10 co-cited references

| Rank | Cited reference | DOI | Citations |
| --- | --- | --- | --- |
| 1 | Sperandio S, 2000, Proc Natl Acad Sci U S A | 10.1073/pnas.97.26.14376 | 174 |
| 2 | Sperandio S, 2004, Cell Death Differ | 10.1038/sj.cdd.4401465 | 139 |
| 3 | Lee D, 2016, Pharmacol Ther | 10.1016/j.pharmthera.2016.01.003 | 97 |
| 4 | Yoon MJ, 2010, Free Radic Biol Med | 10.1016/j.freeradbiomed.2009.12.016 | 69 |
| 5 | Wang WB, 2012, J Cell Physiol | 10.1002/jcp.22956 | 59 |
| 6 | Yoon MJ, 2014, Cell Death Dis | 10.1038/cddis.2014.85 | 50 |
| 7 | Fontana F, 2020, Biochim Biophys Acta Rev Cancer | 10.1016/j.bbcan.2020.188338 | 47 |
| 8 | Yoon MJ, 2014, Oncotarget | 10.18632/oncotarget.2256 | 45 |
| 9 | Yoon MJ, 2012, Cancer Lett | 10.1016/j.canlet.2012.05.018 | 44 |
| 10 | Bury M, 2013, Cell Death Dis | 10.1038/cddis.2013.85 | 42 |

**Table S4.** Top 20 most frequent keywords

| Rank | Keywords | Occurrences | Total link strength |
| --- | --- | --- | --- |
| 1 | paraptosis | 220 | 1428 |
| 2 | apoptosis | 191 | 1371 |
| 3 | cell death | 141 | 978 |
| 4 | autophagy | 96 | 734 |
| 5 | endoplasmic reticulum stress | 94 | 627 |
| 6 | inhibition | 72 | 481 |
| 7 | cancer | 53 | 294 |
| 8 | endoplasmic reticulum | 41 | 315 |
| 9 | human | 41 | 463 |
| 10 | activation | 37 | 253 |
| 11 | cancer cells | 37 | 285 |
| 12 | in vitro | 35 | 256 |
| 13 | ferroptosis | 33 | 295 |
| 14 | mitochondria | 32 | 253 |
| 15 | ROS | 32 | 237 |
| 16 | mechanisms | 31 | 203 |
| 17 | oxidative stress | 31 | 237 |
| 18 | programmed cell death | 31 | 178 |
| 19 | anticancer | 30 | 218 |
| 20 | expression | 29 | 145 |

**Table S5.** Similarities and differences between paraptosis and other forms of cell death

| Form of cell death | Similarities | Key differences | Relationship | Representative references |
| --- | --- | --- | --- | --- |
| Apoptosis | Both belong to programmed cell death. | Apoptosis is typically characterized by caspase activation, DNA fragmentation, nuclear condensation, and apoptotic body formation. Paraptosis is mainly characterized by cytoplasmic vacuolation and ER and/or mitochondrial swelling, and lacks typical apoptotic features. | Paraptosis is often phenotypically identified through comparison with classical apoptosis and may serve as an alternative death route when apoptosis is limited. In some contexts, the two may also occur simultaneously. | (2, 3, 30) |
| Autophagy | Both may show vacuole-like structures. | Autophagy-related vacuoles are mainly associated with double-membrane autophagosomes, autolysosomes, and lysosome-dependent degradation. Paraptosis-associated vacuoles mainly arise from ER and/or mitochondrial dilation and usually do not depend on the classical autophagic degradation program. | In some studies, autophagy and paraptosis may coexist, and paraptosis-related processes may also be accompanied by changes in autophagy-related proteins. | (18, 31, 32) |
| Ferroptosis | Both may be associated with oxidative stress and are regarded as potential alternative death routes for overcoming apoptosis evasion in cancer therapy. | Ferroptosis is mainly driven by disruption of iron and lipid metabolism homeostasis and iron-dependent lipid peroxidation, and is typically characterized by mitochondrial shrinkage, increased membrane density, and reduced cristae. Paraptosis is mainly associated with proteostasis imbalance, ER stress, and mitochondrial dysfunction, and is typically characterized by cytoplasmic vacuolation and ER and/or mitochondrial swelling. | Both may be associated with ROS and MAPK signaling pathways, and may share some stress-related regulatory factors or be co-induced. | (33–40) |
| Necroptosis | Both are non-apoptotic programmed cell death modalities. | Necroptosis depends on the RIPK1/RIPK3/MLKL signaling axis and is characterized by loss of cell membrane integrity. Paraptosis does not depend on this axis and is mainly characterized by cytoplasmic vacuolation and organelle swelling. | The two may co-occur in specific experimental contexts. | (35, 41, 43) |
| Pyroptosis | Both are non-canonical forms of cell death and may participate in antitumor responses. | Pyroptosis depends on inflammasome activation, gasdermin cleavage, membrane pore formation, and inflammatory mediator release. Paraptosis lacks these inflammatory pore-forming features. | Potential interactions may exist in specific antitumor contexts. | (35, 42, 44) |

Abbreviations: ER, endoplasmic reticulum; ROS, reactive oxygen species; MAPK, mitogen-activated protein kinase; RIPK1, receptor-interacting serine/threonine-protein kinase 1; RIPK3, receptor-interacting serine/threonine-protein kinase 3; MLKL, mixed lineage kinase domain-like pseudokinase.
